# Evaluation of Vertical Discrepancies in Crown Seating Using Different Glass Ionomer Cement Volume: An In Vitro Study

**DOI:** 10.7759/cureus.59928

**Published:** 2024-05-08

**Authors:** Sandip Rajput, Mahesh Gandhewar, Sneha Raul, Amruta Bhalerao, Priyadarshani Baisane, Akshay Gandhi

**Affiliations:** 1 Department of Prosthodontics, Guru Gobind Singh College of Dental Sciences and Research Centre, Burhanpur, IND; 2 Department of Prosthodontics, Annasaheb Chudaman Patil Memorial (ACPM) Dental College, Dhule, IND; 3 Dentistry, Gandhi's Dental and Implant Clinic, Baramati, IND

**Keywords:** glass ionomer cement, luting agent volume, gic, post-cementation, pre-cementation, vertical marginal discrepancies

## Abstract

Background

This in vitro study aimed to assess the vertical disparities in the positioning of complete crown castings when different quantities of cement were used and to determine the optimal amount of cement for cementation while minimizing any marginal discrepancies.

Methodology

A total of 60 ideal nickel-chromium (Ni-Cr) crown castings were divided into three groups of experimental volumes of glass ionomer cement, with 20 castings in each group. Group I had completely filled volume with cement, group II had it half-filled, and group III had brushed up cement internally. The crowns were cemented by applying a static load of 5 kg to the cementation apparatus for 10 minutes. The marginal discrepancy between the die and the castings was measured pre-cementation and post-cementation using image analysis software in combination with a stereomicroscope (Motic, USA) at predetermined points that were marked on the die. Statistical analysis was performed using Statistical Package for the Social Sciences (IBM SPSS Statistics for Windows, IBM Corp., Version 16, Armonk, USA) software. A one-way analysis of variance (ANOVA) was used for the intergroup analysis. A paired sample t-test was used for intragroup analysis.

Result

Brushing cement onto the internal surface presented the least mean values (P<0.05) of post-pre-cementation vertical discrepancy (14.92±10.77 μm) when compared to the half-filled cement group (28.42±12.45 μm) and the fully-filled cement group (58.50±20.91 μm).

Conclusion

Cement volume appeared to be a key factor in the vertical marginal discrepancy of the crown. The cement brush applied to the internal surfaces of the crown showed smaller post-cementation vertical discrepancies.

## Introduction

An important factor that ensures the long-term success of fixed prosthodontic restorations is marginal integrity. Accurate marginal adaptation with a minimum discrepancy in the crown is an important goal in prosthodontics. This improves longevity and reduces the risk of restoration misfits associated with periodontal disease and caries [1,2]. At the heart of clinical success for fixed partial dentures lies the luting procedure, where the luting agent serves as a barrier against microbial leakage, sealing the interface between the tooth and restoration and holding them together through some form of surface attachment. This attachment bond may be mechanical, chemical, or a combination of both [3]. The cementation procedure, which influences both the occlusal relationship and marginal fit, underscores the importance of factors that influence crown seating. Methods to facilitate complete seating of crowns are distributed mainly in preparation or casting and modification of the luting procedure by altering the choice of cement, composition of cement, mixing procedure, or cementation load [4-6].

Factors such as the viscosity of the cement, the morphology of the restoration, venting, seating force, and volume of cement may influence the complete seating [7,8]. Some attention has also been given to the amount of cement and how it is applied to achieve better seating. Most researchers have agreed that a smaller volume of cement results in more complete seating [8]. While some have advocated the placement of cement only on the preparation margins, others have suggested brushing up the entire internal surface of the crown [9]. While glass ionomer cement is a commonly used luting agent, there is a scarcity of studies focusing on the seating accuracy of a full-coverage metal crown luted with glass ionomer cement using different cement volumes.

This study aims to delve into the relationship between the vertical seating discrepancy of complete crown casting and the volume of glass ionomer cement employed as a luting agent.

## Materials and methods

**Figure 1:**
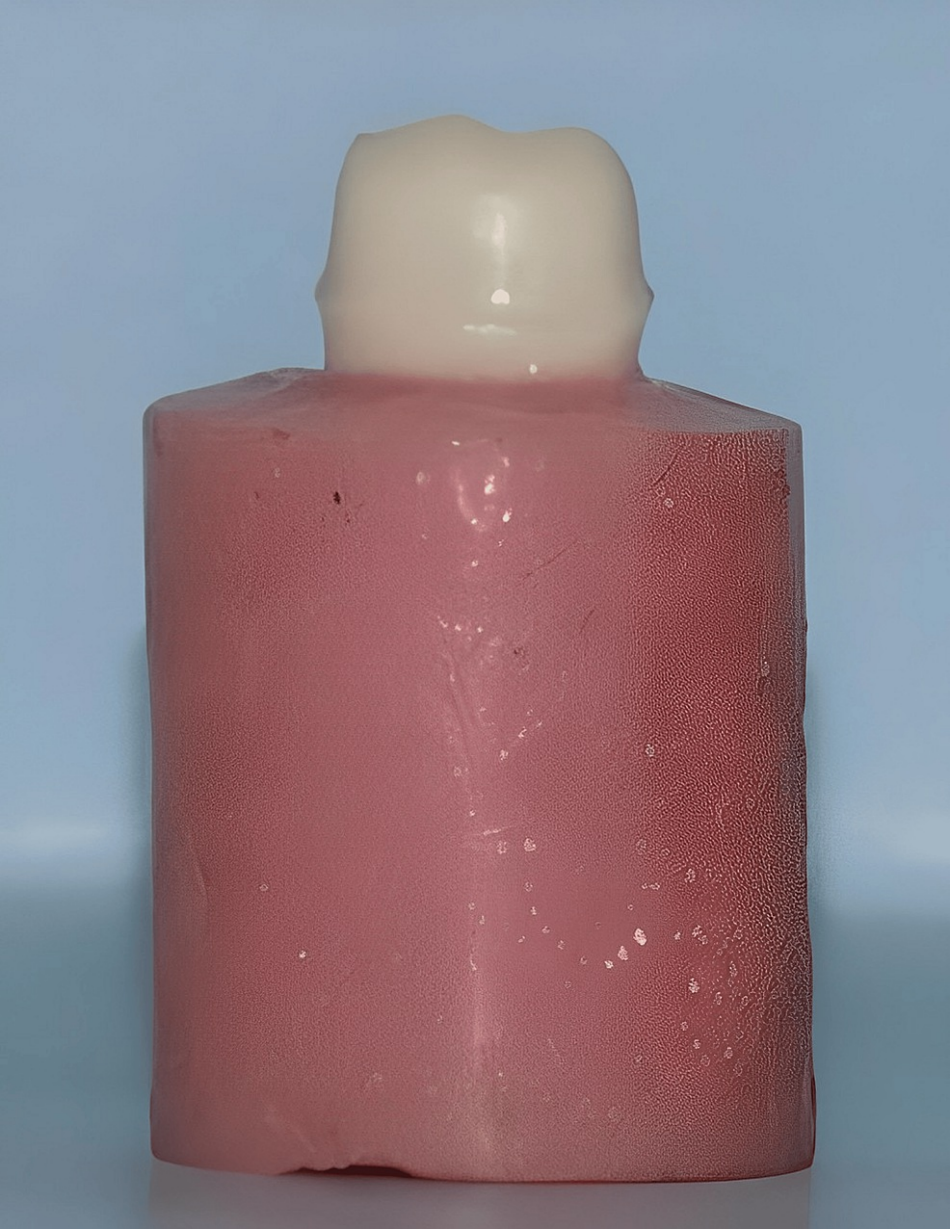
Tooth preparation for full coverage crown

The study utilized an ivorine (Nissan Dental Products Inc., Japan) right mandibular first molar prepared with diamond rotary cutting instruments for a full coverage restoration following the standard recommended procedure (Figure 1).

Light viscosity and putty polyvinyl siloxane (Neopure, Orikam) were used to create an impression mold of the dentoform tooth. This impression mold was poured into Type IV stone (Asian Chemicals, India) to obtain sixty dies. Duplicate dies were poured into Type IV stone for working die fabrication. Wax patterns were fabricated on working dies coated with three coats of die spacer of Pico-Fit (Renfert, Hilzingen, Germany) thickness 40 μm short of 0.5 mm from the margin. Crowns were cast using standard procedure, and the internal fit was verified with a fit-checker. Crown volume was calculated by filling the crown with wax of known density and calculating the volume of wax from its weight and density.

**Figure 2:**
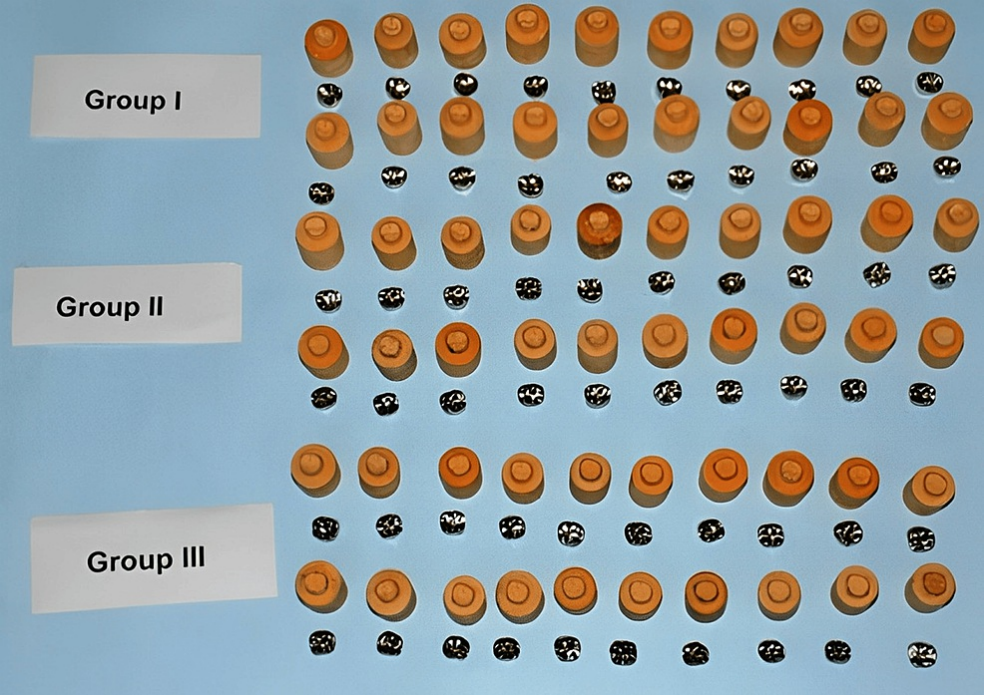
Crowns used in the study

All nickel-chromium (Ni-Cr) complete crown castings (Figure 2) were sequentially seated on the respective die and loaded with a 5 kg weight centered on the crown.

The marginal discrepancy between the die and the castings was measured using image analysis software (Motic Images Plus) in combination with a stereomicroscope (Motic, US) at predetermined points that were marked on the die just near to the margins. Type I glass ionomer cement (Gold label Fuji I, GC Corp.) with a 1.8:1.0 powder-to-liquid ratio was employed for cementation. Standardized cement volume as per Table 1 was dispensed for each group, and crowns were cemented under a static load of 5 kg with cementation apparatus for 10 minutes.

**Table 1:** Standardize glass ionomer cement volume, powder, and liquid dispensed for cementation of crown in each group

Groups	Approximate volume of the crown	Copings (N=60)	Powder dispensed	Liquid dispensed
Group I: Completely filled volume	100%	N=20	2 level scoop (0.36 g)	4 drops (0.20 g)
Group II: Half-filled volume	50%	N=20	1 level scoop (0.18 g)	2 drops (0.10 g)
Group III: Brushed up cement internally	25%	N=20	0.5 scoop (0.09 g)	1 drop (0.05 g)

**Figure 3:**
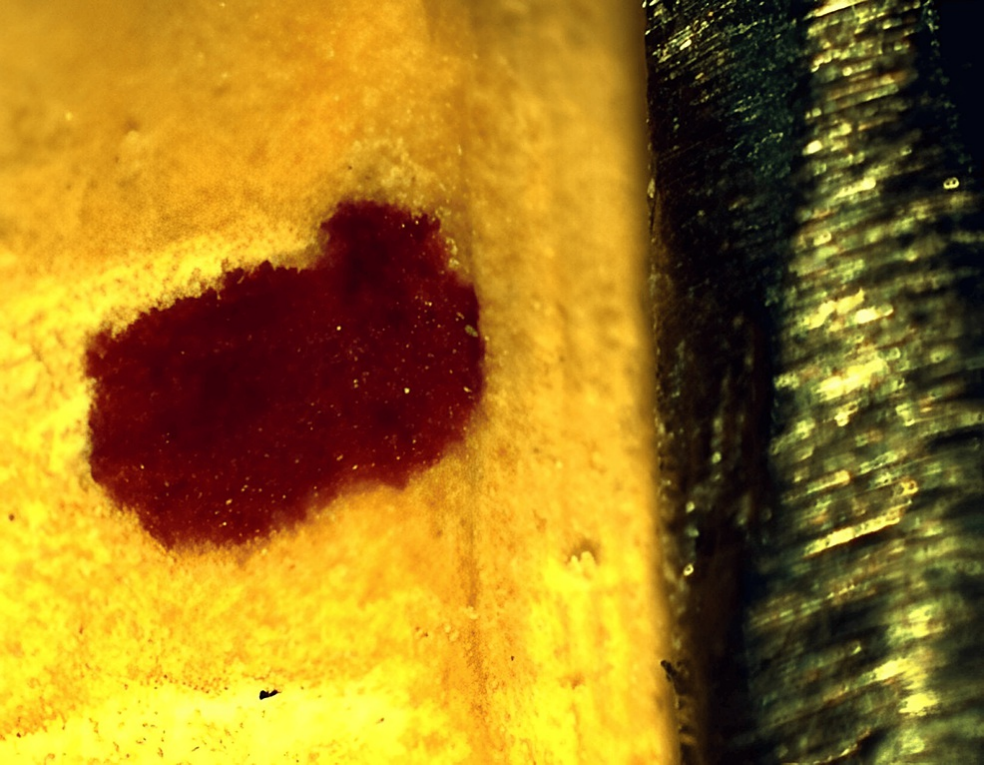
Marginal discrepancy under stereomicroscope

The post-cementation marginal discrepancies of crowns were measured using image analysis software in combination with a stereomicroscope at predetermined points that were marked on the die (Figure 3).

Vertical seating discrepancies in crown seating were determined by the difference between pre- and post-cementation marginal discrepancies measured with a stereomicroscope. Statistical analysis using Statistical Package for the Social Sciences (IBM SPSS Statistics for Windows, IBM Corp., Version 16, Armonk, USA) software included nonparametric tests due to nonparametric normality data (Shapiro-Wilk’s Test). Intergroup analysis employed a one-way analysis of variance (ANOVA), while intragroup analysis used a paired sample t-test, contributing to a comprehensive assessment of the study’s outcome. P (probability) value of less than 0.05 was considered significant in the present study.

## Results

The statistical analysis (one-way ANOVA) demonstrated significant differences (P<0.0001) between the mean post-cementation marginal discrepancies in each group. Brushing cement internally (group III) showed smaller discrepancies compared to other groups. No significant differences (P<0.6) were found in the mean pre-cementation discrepancies (Table 2).

**Table 2:** Vertical marginal discrepancies mean values and SDs for each experimental condition (μm) * means statistically significant

Test Groups	Pre-cementation mean (SD)	Post-cementation mean (SD)	Post- and pre-cementation mean (SD)
Group I: Completely filled volume	94.68 (11.38)	152.45 (24.30)	58.50 (20.91)
Group II: Half-filled volume	89.94 (16.47)	118.36 (19.58)	28.42 (12.45)
Group III: Brushed up cement internally	91.37 (17.07)	106.29 (17.64)	14.92 (10.77)
P value	0.6	0.0001*	0.05*

Multiple comparisons (Tukey's HSD (honestly significant difference)) indicated no significant differences in prevalence discrepancies between groups. However, the post-cementation discrepancy for the filled group differed significantly from the half-filled and brushed-up groups. The half-filled group showed significant differences with the fully-filled group, and the brushed-up group showed no significant differences. The brushed-up group demonstrated significant differences from the fully-filled group and no significant differences from the half-filled group. Post- and pre-cementation vertical discrepancies were statistically significant (P<0.05), with brushing cement onto the internal surface showing the least mean values (14.92±10.77 μm) compared to half-filled (28.42±12.45 μm) and fully-filled groups (58.50±20.91 μm). The comparison of mean marginal discrepancies for buccal, lingual, mesial, and distal surfaces before and after cementation in each group was statistically significant.

Marginal discrepancies in surfaces Tukey's HSD revealed no significant differences among the preparation surfaces of multiple groups when compared with each other except the distal surface comparison of group I and group II, which showed a significant difference. Tukey's HSD revealed significant differences among post-cementation buccal surfaces of multiple group comparisons, except buccal surface comparisons of groups II and III. Tukey's HSD revealed no significant differences among post-cementation lingual surfaces of multiple group comparisons except for the lingual surface comparisons of groups I and III, which showed a significant difference. Tukey's HSD revealed no significant differences among post-cementation mesial surfaces of multiple groups. Tukey HSD revealed highly significant differences among post-cementation distal surfaces in multiple comparisons, except for the distal surface comparison of groups II and III, which showed no significant difference (Tables 3-4).

**Table 3:** Multiple comparisons of pre-cementation surfaces (Tukey's HSD (honestly significant difference))

Dependent Variable	(I) Groups	(J) Groups	Mean Difference (I-J)	Standard Error	Significance
Buccal Pre-cementation	Group I	Group II	12.49000	9.80355	0.416
		Group III	7.11950	9.80355	0.749
	Group II	Group I	-12.49000	9.80355	0.416
		Group III	-5.37050	9.80355	0.848
	Group III	Group I	-7.11950	9.80355	0.749
		Group II	5.37050	9.80355	0.848
Lingual Pre-cementation	Group I	Group II	-4.20500	10.02535	0.908
		Group III	4.01250	10.02535	0.916
	Group II	Group I	4.20500	10.02535	0.908
		Group III	8.21750	10.02535	0.692
	Group III	Group I	-4.01250	10.02535	0.916
		Group II	-8.21750	10.02535	0.692
Mesial Pre-cementation	Group I	Group II	-13.65000	10.43850	0.397
		Group III	-14.04000	10.43850	0.377
	Group II	Group I	13.65000	10.43850	0.397
		Group III	-.39000	10.43850	0.999
	Group III	Group I	14.04000	10.43850	0.377
		Group II	.39000	10.43850	0.999
Distal Pre-cementation	Group I	Group II	24.31500	8.81317	0.021
		Group III	16.16000	8.81317	0.168
	Group II	Group I	-24.31500	8.81317	0.021
		Group III	-8.15500	8.81317	0.627
	Group III	Group I	-16.16000	8.81317	0.168
		Group II	8.15500	8.81317	0.627

**Table 4:** Multiple comparisons of post-cementation surfaces (Tukey's HSD (honestly significant difference))

Dependent Variable	(I) Groups	(J) Groups	Mean Difference (I-J)	Standard Error	Significance
Buccal Post-cementation	Group I	Group II	46.16200	13.95403	0.005
		Group III	41.65200	13.95403	0.011
	Group II	Group I	-46.16200	13.95403	0.005
		Group III	-4.51000	13.95403	0.944
	Group III	Group I	-41.65200	13.95403	0.011
		Group II	4.51000	13.95403	0.944
Lingual Post-cementation	Group I	Group II	35.52000	15.31423	0.061
		Group III	66.25500	15.31423	0.000
	Group II	Group I	-35.52000	15.31423	0.061
		Group III	30.73500	15.31423	0.120
	Group III	Group I	-66.25500	15.31423	0.000
		Group II	-30.73500	15.31423	0.120
Mesial Post-cementation	Group I	Group II	18.66200	14.22172	0.394
		Group III	33.37500	14.22172	0.057
	Group II	Group I	-18.66200	14.22172	0.394
		Group III	14.71300	14.22172	0.558
	Group III	Group I	-33.37500	14.22172	0.057
		Group II	-14.71300	14.22172	0.558
Distal Post-cementation	Group I	Group II	38.91800	10.29397	0.001
		Group III	46.28300	10.29397	0.000
	Group II	Group I	-38.91800	10.29397	0.001
		Group III	7.36500	10.29397	0.755
	Group III	Group I	-46.28300	10.29397	0.000
		Group II:	-7.36500	10.29397	0.755

## Discussion

Significant differences were observed among the groups after cementation, suggesting that cement volumes do play a crucial role in post-cementation marginal discrepancies. However, it's noteworthy that no significant differences were found in pre-cementation marginal discrepancy values, highlighting the precision of the baseline fabrication processes by conventional process across the groups.

The study highlights the impact of cement volumes on the marginal discrepancies of the evaluated groups and correlates well with findings from other in vitro studies on crown discrepancies after cementation [8-11]. Notably, the literature lacks studies specifically comparing different glass ionomer cement volumes for the cementation of metal crowns, making this study a valuable addition to the existing knowledge. The standardized tooth preparation with a chamfer finish line and 6° total taper aimed to resemble the clinical situation. However, the 6° taper angle used in this study may not fully replicate clinical conditions, as the clinical taper typically varies between 12° and 20°. Despite this limitation, the results are consistent with previous studies using similar taper angles.

The application of three layers of die spacer (40 μm) aimed to provide internal relief while accommodating the cement layer and irregularities on the tooth and inner crown surface [12-16]. Pre-cementation vertical marginal discrepancies fell within clinically acceptable limits between 100 and 120 μm, with mean values aligning with previous studies [11,17-20]. The choice of glass ionomer cement as the luting agent was based on its favorable clinical performance, exhibiting attributes such as compressive strength, fluoride ion release, and a low coefficient of thermal expansion. It has a low film thickness and maintains a relatively constant viscosity for a short time after mixing. This results in improved seating of cast restoration compared with zinc phosphate cement [21].

This study utilized a standardized load from the above position of 5 kg with a cementation apparatus for 10 minutes. To ensure the complete setting of the luting agent and prevent rebound, the glass ionomer cement setting time was exceeded by five minutes and 30 seconds. Rebound refers to the possibility of the luting agent, such as glass ionomer cement, partially regaining its original shape or position after compression during the setting process. This can occur if the setting time is not allowed to be fully complete before removing the applied load or pressure. By extending the setting time by five minutes and 30 seconds, the study aims to ensure the complete setting of the luting agent, thereby reducing the risk of rebound and achieving optimal bonding or cementation results. The selected load aligns with prior research by Jorgensen and Petersen (1963), indicating improved crown seating with increased force up to 5 kg (49 N), beyond which the gains were marginal. The post-cementation marginal discrepancies increased after cementation in all groups, with significant differences observed. The study emphasizes the complexity of seating crowns, considering factors like intra-coronal pressures and the hydrodynamic situation during cementation. The research aligns with the consensus that a marginal gap between 100 and 120 μm is clinically acceptable, and the post-cementation discrepancies within this range for brush-up and half-filled groups are noteworthy [11,17-20]. 

For this in vitro evaluation, differences were observed between buccal, lingual, mesial, and distal surfaces, and although this could be due to the more complex geometric form of restoration resulting in fit problems, previous studies [22,23] have reported similar results in individual ceramic crowns. Some studies done by Kokubo et al. [24] and Holden et al. [25] have evaluated the marginal discrepancies without taking the cementation process into consideration. Evaluating discrepancies without luting them is not reflective of clinical reality because the cement and the cementation process play a relevant role in the final discrepancy achieved. In the current study, although space was created to allow the cement to flow into the space between the tooth and internal surface, the results for the metal crown and cement combinations increased the discrepancy after cementation.

Our study revealed that crowns with larger cement volumes exhibited higher post- and pre-cementation marginal discrepancies. This could be attributed to several factors: entrapment of cement on the occlusal surface of the abutment, sealing the natural escape route during crown seating, hydraulic pressure during cementation leading to filtration along narrow spaces between crown and abutment, hindering complete seating by slowing cement escape, and increased viscosity of cement over time as greater volumes took longer to escape, impeding complete seating. The initial rapid seating of crowns may be due to the initial low viscosity of the luting agent, which increased over time along with hydraulic pressure buildup, limiting cement escape. Completely filled crowns (group I) experienced the greatest seating discrepancy (58.50±20.91 µm) as the large initial cement volume hindered further flow before viscosity and pressure increased. Tilt may have also contributed to seating discrepancies, particularly in group I, where crowns were difficult to align onto the abutment. In group III, where brush-up cement volume was used, better alignment onto the abutment was observed initially, resulting in minimal seating discrepancies (14.92±10.77 µm). Despite a slight increase in marginal discrepancy post-cementation, the predetermined internal spaces of 40 μm for cement appeared sufficient to accommodate the film thickness, leading to no or minimal increase in marginal fit. Compared to a previous study by Tan and Ibbetson [8], our study showed lower post- and pre-cementation discrepancies (14.9 µm-58.5 µm), likely due to differences in luting agents and measurement methods.

The limitations of the research, particularly its in vitro nature, are acknowledged. Simulating the complex clinical oral environment with variations in tooth preparations, finish line designs, and intraoral conditions remains a challenge. The focus on vertical marginal gaps and the absence of quantification for horizontal relationships are identified as limitations, emphasizing the potential influence of over- or under-contouring on plaque accumulation and gingival irritation.

## Conclusions

In conclusion, this study focuses on the critical role of glass ionomer cement volume in minimizing vertical discrepancies during crown cementation. The results emphasize the need for careful consideration of cement application techniques to achieve optimal marginal adaptation, thereby enhancing the longevity and stability of dental restorations. Additionally, the findings underscore the intricacies of the cementation process, highlighting the importance of meticulous attention to detail in clinical practice. Further research in this area could explore additional factors influencing crown cementation outcomes, ultimately contributing to advancements in dental materials and techniques.
